# Coronaviruses: Molecular and Cellular Biology

**DOI:** 10.2217/17460794.3.2.119

**Published:** 2008-02-29

**Authors:** Dale L Barnard

**Affiliations:** 1 Institute for Antiviral Research, Utah State University, 5600 Old Main Hill, Logan, UT 84322-5600, USA. Tel.: +1 435 797 2696; Fax: +1 435 797 3959; dale.barnard@usu.edu

Coronaviruses cause respiratory and gastrointestinal diseases in various vertebrates, including minor respiratory diseases in humans. Because of that fact, focused research on these viruses was limited to a few dedicated, but talented, research groups. However, that changed in 2003, when a new coronavirus emerged, designated as severe acute respiratory syndrome corona-like virus (SARS-CoV), which caused a life-threatening respiratory disease characterized as an acute respiratory distress syndrome with a fatality rate of almost 10%. Suddenly, the world’s researchers devoted countless hours to attempting to identify, characterize and develop treatments for this new virus. Knowledge of coronavirus virology multiplied exponentially and, more importantly, quickly. Within 1 year of discovery, the genome of the virus had been sequenced and a great deal was known about its replication cycle. Unfortunately, discovery of efficacious treatments has not developed as quickly. In addition, and probably as a result of the emphasis on SARS-CoV research, new coronaviruses causing less severe, yet significant, disease in humans have also been discovered.

Some 4 years after the outbreak of SARS, this book edited by Volker Thiel tries to summarize the most recent advances in coronavirus molecular biology, vaccine development and coronavirus therapeutics, with great emphasis on SARS-CoV. The authors of most of the chapters are some of the most respected experts in the field of coronavirus research. The book consists of 16 chapters divided into two parts. The first section contains chapters that discuss the evolution and molecular biology of the *Coronaviridae*, often using information gained from studying group 2 coronaviruses. The second part of the book consists of chapters that review the pathogenesis of coronavirus diseases, such as those caused by SARS-CoV, feline infectious peritonitis virus (FIPV), mouse hepatitis virus (MHV) and human NL63 coronavirus (HCoV-HL-63). Also included are chapters on immune responses to coronaviruses, vaccine development and the current status of anti-coronavirus therapies.

The book represents a valiant effort to summarize the recent advances in coronavirus research – an extremely fast moving area of study – by some of the world’s leading experts in coronavirus virology. The knowledge contained in the book demonstrates how quickly and relatively efficiently the worldwide research community can meld together into a giant collaborative effort and concentrate its resources to rapidly begin to understand and solve problems, in this case a health problem.

## Chapter 1

### ‘Coronavirus Binding and Entry’

This chapter, authored by David Wentworth and Kathryn Holmes, is a review of the initial stages of coronavirus infection, binding and entry. The authors present several models of binding and attachment to cells by coronaviruses. Much of the information presented comes from studies of SARS-CoV. This chapter is an excellent generalized review of the coronavirus proteins necessary for attachment and the receptors to which coronaviruses attach.

## Chapter 2

### ‘The Coronavirus Replicase Gene: Special Enzymes for Special Viruses’

John Ziebuhr and Eric Snijder are the authors of this chapter. This chapter is a review of the information known regarding expression of the replicase gene complex of coronaviruses and how the proteins of this complex function. Included in the review are discussions of the functions of RdRp polymerase, exo- and endoribonucleases, and the proteases associated with the replication complex. The authors do an excellent job in presenting what is known about coronavirus RNA replication, relying heavily on data gathered from experimentation with MHV, one of the most intensely studies viruses of the *Coronaviridae*. Also included are studies of other members of the *Nodovirales* that support the various proposed mechanisms of coronavirus RNA replication.

## Chapter 3

### ‘Genomic *Cis*-acting Elements in Coronavirus RNA Replication’

This chapter by Paul Masters reviews studies of the 3´ and 5´ and internal genomic *cis*-acting elements associated with coronavirus RNAs. The author makes a good effort to summarize the potential mechanisms whereby *cis*-acting RNA elements act to promote coronavirus gene replication. He makes excellent use of data, mostly from the study of MHV replication, to support some of the proposed mechanisms of action for these *cis*-acting elements.

## Chapter 4

### ‘Coronavirus RNA Synthesis: Transcription’

Luis Enjuanes, Isabel Sola, Sonia Zuñiga and José L Morena author this chapter on the mechanism of RNA transcription in coronaviruses. The authors present the most widely accepted model of coronavirus transcription, summarized by them diagrammatically as a ‘three-step working model’. The authors also present three alternative models, one of which relies on information gathered from studying arteriviruses. The authors discuss in copious detail the many cellular and viral factors involved in coronavirus transcription and end the chapter with a brief discussion on *Nodovirales* evolution based on transcription strategies. Although this chapter very thoroughly reviews the topic of transcription, there are a few noticeable typos and one or two awkwardly written sentences.

## Chapter 5

### ‘Reverse Genetic Analysis of Coronavirus Replication’

Volker Thiel covers a very important topic on the development of reverse genetics SARS-CoV, which has fostered astoundingly rapid advances in the understanding of SARS-CoV replication. The author selects seven applications for which reverse genetics has been used to study recombinant viruses and various gene products, with emphasis on SARS-CoV. He also discusses the development of coronavirus-based vectored RNAs and the generation of coronavirus vaccines using virus-like particles. A very well-explained figure portraying an HCoV-229E-based replicon accompanies this chapter.

## Chapter 6

### ‘Coronavirus Genome Selection and Packaging’

The authors, Krishna Narayanan and Shinj Makino, review the current understanding of the initial stages in coronavirus particle assembly. The authors describe the various virion MHV packaging signal systems about which most is known, as well as those packaging systems associated with infectious bronchitis virus (IBV), transmissible gastroenteritis virus (TGEV) and SARS-CoV. The last section discusses *trans*-acting factors involved in coronavirus packaging, primarily using data from MHV experimentation. This chapter also includes a brief, yet informative, summary of the various packaging signals that are discussed.

## Chapter 7

### ‘Molecular Evolution of Group 2 Coronaviruses’

This chapter contains a very good summary of the data discussed by the authors, Leen Vijgen, Els Keyaerts and Marc Van Ranst. In this chapter, the authors review data demonstrating the molecular evolution of the MHV group 2 coronaviruses (MHV 1–3, A59, JHM and MHV-S) and the bovine coronavirus (BCoV)-related strains, BCoV, human coronavirus OC43 (HCoV-OC43), porcine hemagglutinating myelo-encephalitis virus (PHEV), canine respiratory coronavirus (CRCoV) and equine coronavirus (ECoV). The authors provide the reader with a standard phylogenetic tree with a rather lengthy figure legend, perhaps too long. The information provided in the chapter is an informative review of the mechanisms that drive virus evolution in general, along with an explanation of the evolutionary history of the group 2 viruses.

## Chapter 8

### ‘Avian Coronavirus Diseases and IBV Vaccine Development’

Paul Britton and Dave Cavanagh discuss developments and future strategies in the development of IBV vaccines. IBV is extremely important because it causes major losses in the poultry industry. The authors first outline the natural course of coronavirus disease in poultry and then discuss the devastating potential for these viruses to infect other domestic and wild avian species. The authors also provide reasons for the lack of efficacy of current vaccines: multitudinous serotype variation without cross-protection and no induction of long-term immunity. The authors report that small differences (<5%) in amino acid sequences in the major immunologically recognized epitopes can lead to lack of cross-protection between serotypes. The authors also review the nature of the protective immune response that a vaccine should elicit and compare the efficacy of attenuated and killed vaccines in eliciting such responses. They review the process of virus attenuation and report on two, yet to be licensed, experimental vaccine approaches for coronaviruses: subviral vaccines and adenovirus-vectored vaccines. *In ovo* vaccination is also discussed as a future method of immunizing poultry.

## Chapter 9

### ‘Feline Coronaviruses: A Tale of Two-Faced Types’

The authors of this chapter are Bert Jan Haijema, Peter Rottier and Raoul de Groot. These authors discuss the pathogenesis and natural infection history of feline coronavirus (FCoV) infections in domestic cats, including enterovirus infections caused by enteric FCoV (eFCoV) and infectious peritonitis caused by FIPV and sometimes by eFCoV. Also included in the chapter are sections on the molecular biology of these viruses, and the potential for vaccine development. The authors compare and contrast the relatively benign eFCoV with the highly pathogenic form of FIPV. The remainder of the chapter describes in great detail the molecular biology of the highly pathogenic FIPV WSU strains.

## Chapter 10

### ‘Control of Neurotropic MHV by Multifactorial Mechanisms’

In this chapter, written by Cornelia Bergman and Stephen Stohlman, the authors review the mechanisms involved in the establishment of two types of brain infections caused by neurotropic MHV. They also discuss immune system factors that contribute to acute and persistent infections caused by MHV. The authors then explain the mechanism of sustained antibody secretion within the CNS that is necessary for prevention of virus recrudescence from persistently infected cells: the maintenance of virus-specific antibody in the CNS.

## Chapter 11

### ‘SARS Coronavirus: Pathogenesis and Correlation with Clinical Disease’

John Nichols and Malik Peiras describe the pathogenesis of SARS-CoV infections in humans and suggest that the pathogenesis is mainly due to damage to type I pneumocytes resulting in diffuse alveolar damage and loss of function, leading to respiratory distress. They also describe the clinical picture and outcome of the disease known as SARS and discuss the possible deleterious effects of the hyperimmune response to the virus, which is covered in slightly greater detail in the next chapter. They present a very good review of what is known about the pathogenesis of SARS infections as of early 2007.

## Chapter 12

### ‘SARS Coronavirus and the Antiviral Cytokine Response’

Perhaps a more appropriate title for this chapter would be ‘Interferon Responses to SARS Infection’, because the authors, Martin Spiegel and Friedermann Weber, spend most of the chapter reviewing interferon (IFN) responses as a consequence of SARS-CoV infection. They begin with a general, detailed review of the classical IFN-induction pathways. They then discuss how SARS-CoV initiates infection despite the activation of IFN-induction pathways, using some of their data to show that SARS-CoV counteracts IRF-3 by sending it back to the cytoplasm from the nucleus, where it acts to inactivate the IFN-β promoter. The authors also discuss the role of IL-8 in reducing IFN responses, which is produced in significant amounts during a SARS infection. The authors also speculate on how SARS-CoV infection induces the so-called ‘cytokine storm’ associated with SARS infections. It would have been appropriate if the authors had included in their review a discussion of the deregulation of the cytokine response in the lungs, where much of the SARS infection occurs and its relationship to the systemic cytokine response as detected in serum.

## Chapter 13

### ‘Grand Challenges in Human Coronavirus Vaccine Development’

Barry Rockx and Ralph Baric begin this chapter by introducing the reader to the human coronaviruses that cause disease. They then describe the genetics of SARS-CoV and the functions of some of the gene products. They also provide an interesting discussion of the evolution of SARS-CoV, the pathogenesis of the infection caused by SARS-CoV, the immune response to the virus and the components of the immune system that are protective against the infection caused by SARS-CoV. To enable the reader to quickly review all of the vaccines and vaccine trials published to date, the authors insert a comprehensive table of SARS-CoV vaccines tested as of early 2007 and the results of any challenge studies done with the vaccines listed. They also discuss the future development of live-attenuated vaccines and replicon particle vaccines. They summarize the challenges of developing a successful, protective coronavirus vaccine, especially for SARS-CoV. They suggest that such a vaccine must elicit broad-spectrum cross-reactivity to all the potential quasi-species that will probably arise and that coronavirus vaccines must not trigger an immune enhancement of the challenge virus. This section is well written and very thorough.

## Chapter 14

### ‘SARS and the Other “New” Coronaviruses’

In this chapter, Leo Poon discusses the emergence of human coronaviruses, with special emphasis on the emergence of SARS-CoV and its origins. The author briefly reviews the epidemiological history of the SARS epidemics and discusses the basis of the classification of SARS-CoV within the *Coronaviridae*. He also devotes several pages to the clinical and molecular diagnostic tests available for detection of SARS-CoV. There are also sections in which the novel group 3 coronaviruses of birds and dogs and newly recognized human pathogens, HCoV-NL63 and HCoV-HuK1, are briefly discussed. HCoV-NL63 and the infection that it causes are described in more detail in the next chapter.

## Chapter 15

### ‘Human Coronavirus NL63, a Long Lost Brother’

This chapter, authored by Krzysztof Pyrc and Lia van der Hoek, reviews what is known about the novel group I coronavirus HCoV-NL63 (NL63). The authors review the discovery and identification of HCoV-NL63, the molecular biology of the virus, including genomic organization, the transcription and translation strategies leading to virus replication, post-translational modifications of virus proteins and the known functions of the virus proteins. There is also an entire section on virus attachment and entry into the host cell. The epidemiology of HCoV-NL63 is reviewed, and the authors discuss the concept of HCoV-NL63 as a coinfecting agent in many respiratory diseases. They postulate the significance of coinfection with HCoV-NL63, especially in relation to the finding that coinfection with HCoV-NL63 often results in more hospitalization of patients than do respiratory infections caused by a single agent. A paragraph is devoted to the putative association of HCoV-NL63 with Kawasaki disease or lack thereof. In the final section, the authors describe potential antiviral targets in the viral replication cycle and review the few chemotherapy studies that have been carried out on treatment of HCoV-NL63 infections.

## Chapter 16

### ‘Current Status of Antiviral Severe Acute Respiratory Syndrome Coronavirus Research’

The authors, Els Keyaerts, Leen Vijgen and Marc Van Ranst, briefly review the potential antiviral targets on the SARS virion and in the virus replication cycle. They provide the reader with a simple yet effective cartoon of the virus replication cycle to illustrate sections of the virus replication cycle that could be targeted by antiviral chemotherapies. Also included in this chapter is a comprehensive list of compounds and other materials that have been reported to show efficacy against SARS-CoV *in vitro*. This leads to the discussion of some of the pitfalls of this chapter. In many cases compounds found in the table have been demonstrated to have *in vitro* efficacy by some laboratories and not to have *in vitro* efficacy in other laboratories. The authors should have discussed the problem of laboratory-to-laboratory variability in determining efficacy of compounds against SARS-CoV. The authors, when reporting on compound efficacy against SARS-CoV, many times forget to report on the selectivity of the compound. Even though some of the compounds described are potent inhibitors of SARS-CoV, they are toxic enough not to be selective inhibitors. Selectivity should have been emphasized in a more critical review of the compounds listed in their table, because compounds with selectivity indices of less than 20 often do not show activity in animal models. A number of the compounds mentioned in the table fall into this category. The reader is also not told whether or not the activity is virucidal; however, that is often the fault of the group publishing the data. Virucides are much less likely to be efficacious in treating lung infections owing to the toxicity often associated with them, lack of oral bioavailability, and the lack of efficacy against cell-to-cell transmission of virus. The authors fail to mention the few studies that have been published on the activity of several of these compounds in a mouse lung replication model. A number of the active and inactive compounds mentioned in the table do not prevent virus replication in the lungs of mice. For example, ribavirin has been found not only to have poor selectivity *in vitro*, but actually to prolong and enhance viral replication in a mouse lung replication model; a finding that has since been verified in a newly established mouse adapted SARS-CoV lethal murine model. Overall, the authors should be more critical in their review of the antiviral agents that have purported activity against SARS-CoV *in vitro*.

## Conclusion

The book entitled *‘Coronaviruses: Molecular and Cellular Biology’* presents a comprehensive review of the field of coronavirus virology. The contributors to the book are many of the experts in coronavirus virology. The contents of the book are particularly useful for those wishing to conduct research on coronaviruses because most of the authors include in their summary or conclusion paragraphs a number of key questions or problems for which research needs to be carried out to further the understanding of coronaviruses and how to treat the infections they cause. Most of the figures are very useful in helping the reader understand the narratives provided by the authors. The few tables provided are excellent in summarizing information that could be useful to readers wanting to know, for example, what antiviral agents have been found active against SARS-CoV *in vitro* or what vaccines have been tested against SARS-CoV.

There are some deficiencies to be found in the book, as there are in any comprehensive review. One minor problem, of course, is that the information is dated, but this book is more current than most review books. Perhaps the most problematic part of the book is the last chapter. The authors of the last chapter should be more critical on reporting of the compounds with published activity against SARS-CoV. Many of the compounds have been found active only in one laboratory but not in another and many have very poor selectivity, but this is not emphasized. Another small problem that the editor could have resolved is the standardization of the acronym for severe acute respiratory syndrome corona-like virus. Several acronyms for this virus were used throughout the text. Most experts in the field, including taxonomists, prefer the term SARS-CoV. A book such as this could establish that this acronym be used.

When reading the book, the reader should be aware that contents are current as of early 2007 and that since then some further advances have been made in the molecular biology of SARS-CoV. However, this text is recommended for those who want to quickly be brought up to date on coronavirus research or who want to know what research questions remain to be answered in the field of coronavirus virology.

